# Males develop faster and more severe hepatocellular carcinoma than females in *kras*^*V12*^ transgenic zebrafish

**DOI:** 10.1038/srep41280

**Published:** 2017-01-24

**Authors:** Yan Li, Hankun Li, Jan M. Spitsbergen, Zhiyuan Gong

**Affiliations:** 1 Department of Biological Sciences, National University of Singapore, 117543, Singapore; 2 Department of Microbiology, Oregon State University, Corvallis, Oregon, 97331, USA

## Abstract

Hepatocellular carcinoma (HCC) is more prevalent in men than women, but the reason for this gender disparity is not well understood. To investigate whether zebrafish could be used to study the gender disparity of HCC, we compared the difference of liver tumorigenesis between female and male fish during early tumorigenesis and long-term tumor progression in our previously established inducible and reversible HCC model – the *kras*^*V12*^ transgenic zebrafish. We found that male fish developed HCC faster than females. The male tumors were more severe from the initiation stage, characteristic of higher proliferation, activation of WNT/β-catenin pathway and loss of cell adhesion. During long-term tumor progression, the male tumors developed into more advanced multi-nodular tumors, whereas the female tumors remain uniform and homogenous. Moreover, regression of male tumors required longer time. We further investigated the role of sex hormones in *kras*^*V12*^ transgenic fish. Estrogen treatment showed tumor suppressing effect during early tumorigenesis through inhibiting cell proliferation, whereas androgen accelerated tumor growth by promoting cell proliferation. Overall, our study presented the zebrafish as a useful animal model for study of gender disparity of HCC.

Hepatocellular carcinoma (HCC), a leading cause of cancer-related death, occurs more frequently in men than in women in all over the world, with a man: women ratio ranging from 2:1 to 8:1 in different geographic regions^1^. Women tend to have less aggressive liver tumors than men at initial diagnosis^2^,^3^ and also have a better survival than men after treatments^4^,^5^. Possible contributing factors for the gender disparity of HCC include gender-specific lifestyle and social environments, such as alcohol consumption, smoking and HBV or HCV infection. Roles of sex hormones have also been implicated as postmenopausal women seem to be more susceptible to HCC than premenopausal women^1^.

Similar to human, gender difference of HCC is also observed in rodent models, including both genetically or chemically induced HCC^6^,^7^,^8^. Both sex hormones and inflammatory response have been implicated in the gender disparity of HCC. Administration of estrogen or castration inhibited the development of DEN-induced HCC in male mice, whereas testosterone supplement or ovariectomy in female mice increased the susceptibility of HCC^7^. It has been suggested that estrogen-mediated inhibition of IL-6 production by Kupffer cells was the reason for the reduced risk of DEN-induced HCC in female mice^9^. Studies using orthotopic and ectopic mouse models indicated that estrogen could inhibit the alternative activation of macrophage and suppress tumor progression via ERβ (estrogen receptor β) and the Jak1-Stat6 pathway^10^. Animal model studies have also indicated the ability of androgen to enhance the TGFα-related hepatocarcinogenesis and hepatocyte proliferation^11^.

Although the studies from rodent model have provided some insights into the roles of sex hormones in gender disparity of HCC, the tumor-promoting effect of androgen remains controversial and hormone therapy of HCC has led to different clinical outcomes^12^. In addition, many of the animal studies have been conducted by chemical-induced carcinogenesis. The pathogenesis of HCC in these chemically induced models may differ in molecular pathways and may be difficult to reliably compare to human HCC^9^. Another disadvantage of the mouse model is the very long latency period (5–24 months) before the establishment of HCC in either chemical-induced carcinogenesis or genetically engineered mouse models^13^.

Our group has previously established several inducible and reversible HCC models in zebrafish by transgenic expression of selected oncogenes^14^,^15^,^16^,^17^,^18^. Usually homogeneous HCC across the entire liver was developed within one month of oncogene induction. Withdrawal of the chemical inducer caused the suppression of oncogene expression and led to a rapid liver tumor regression. The *kras*^*V12*^ transgenic line was the most potent strain for HCC induction. In the present study, we aim to investigate whether the inducible transgenic zebrafish model could also model the gender disparity of HCC. The difference of liver tumorigenesis between female and male fish was examined during early tumorigenesis and long-term tumor progression using the *kras* transgenic line. We further investigated the role of estrogen and androgen during early tumorigenesis. Overall, our study indicated the zebrafish as a potentially valuable animal model for studying the gender disparity of HCC.

## Results

### Male *kras* ^*V12*^ zebrafish developed HCC faster than female during early tumorigenesis

**Figure 1:**
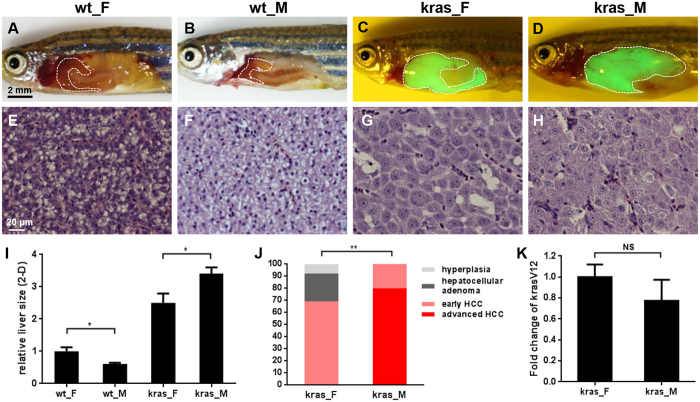
Male *kras*^*V12*^ zebrafish developed HCC faster than female during early tumorigenesis. (**A**–**D**) Representative liver images from female (F) and male (M) wild type (wt) and *kras*^*V12*^ fish (4 months old) after 10 days of doxycycline induction. The livers are outlined in dash lines. (**E**–**H**) Representative liver histology by H&E staining from fish as described in (**A**–**D**). (**I**) 2D liver sizes measured from the left lateral side. The liver size of wild type females (wt_F) is arbitrarily set as 1 and the sizes in other groups are relative to wt_F. (n = 15 for each group, *p < 0.05). (**J**) Quantification of histological phenotypes of livers from female and male *kras*^*V12*^ zebrafish (**p < 0.01). (**K**) Fold change of *kras*^*V12*^ expression in female (set as 1) and male (relative to kras_F) *kras*^*V12*^ fish (NS, not significant). Scale bars, 2 mm for (**A**–**D**) and 20 μm for (**E**–**H**).

To determine the induction time required for HCC formation, different concentrations of doxycycline (5, 10 and 20 mg/L) and induction durations (4, 7 and 10 days) were tested in 4-month-old *kras*^*V12*^ transgenic zebrafish. The earliest onset of histological HCC could be observed after 7 days of induction with 20 mg/L doxycycline and most of the fish developed histological HCC by 10 days post induction. To examine whether there is a gender difference during early liver tumorigenesis in the *kras*^*V12*^ induced zebrafish HCC model, male and female *kras*^*V12*^ zebrafish were treated with 20 mg/L doxycycline for 10 days. Measurement of the two-dimensional liver size from the left lateral side showed that there was prominent liver enlargement in both male and female fish after induction of *kras*^*V12*^ for 10 days (Fig. 1A–D). The livers of induced *kras*^*V12*^ male fish were expanded more significantly than those of *kras*^*V12*^ female, whereas in wild type fish the male livers were only half the size of female livers (Fig. 1I). Histological examination of the livers revealed that all of the induced *kras*^*V12*^ male fish had developed HCC (20% early, 80% advanced), whereas in *kras*^*V12*^ females only 70% had early HCC and the rest 30% had hepatocellular adenoma or hyperplasia (Fig. 1E–H,J). Moreover, most of the male HCC (80%) had reached advanced HCC stage (loss of hepatocyte plate structure, prominent nuclei, nucleus pleomorphism, presence of multiple nucleoli in most of the hepatocytes), whereas the female HCC were all at early HCC stage (loss of hepatocyte plate structure, prominent nuclei, presence of multiple nucleoli in only a few hepatocytes). Thus, there was a faster HCC development during early tumor formation in male *kras*^*V12*^ zebrafish than female *kras*^*V12*^ zebrafish. The difference in HCC development in the two genders was not due to the levels of induced *kras*^*V12*^ expression as both male and female *kras*^*V12*^ transgenic fish showed similar levels of *kras*^*V12*^ induction (Fig. 1K).

**Figure 2:**
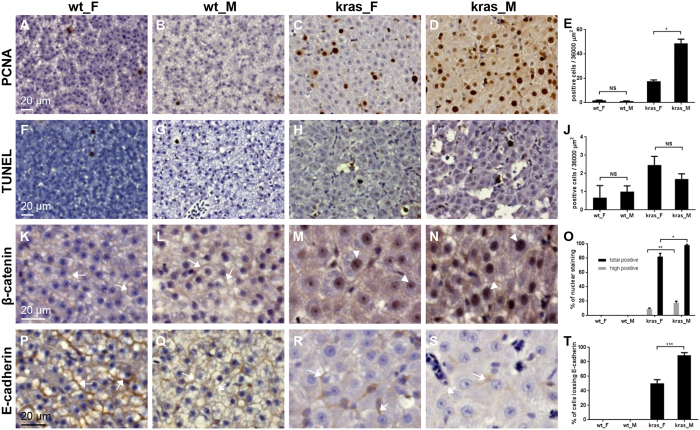
The male tumors were characterized by higher level of cell proliferation, β-catenin activation and E-cadherin loss. Representative immunohistochemical staining of liver sections from female and male wild type and *kras*^*V12*^ fish (4 months old) after 10 days of doxycycline induction are shown. (**A**–**D**) Immunohistochemical staining for PCNA. **(E**) Quantification of PCNA nuclear staining. (**F**–**I**) TUNEL staining for apoptotic cells. (**J**) Quantification of apoptotic cells. (**K**–**N**) Immunohistochemical staining for β-catenin. (**O**) Quantification of β-catenin nuclear staining. (**P**–**S**) Immunohistochemical staining for E-cadherin. (**T**) Quantification of cells losing E-cadherin membrane staining. *p < 0.05; **p < 0.01; ***p < 0.001; NS: not significant. Scale bar, 20 μm for all picture panels. Examples of membrane and nuclear staining are indicated by arrows and arrowheads, respectively.

To further characterize the gender difference during early development of HCC, proliferation and apoptosis within the tumor tissue were examined by PCNA staining and TUNEL assay, respectively (Fig. 2). In normal adult livers, there was a very low level of proliferation as indicated by the rare nuclear staining of PCNA, which was comparable between males and females. In liver tumors, significantly higher level of proliferation was detected in males than in females (Fig. 2A–E). Levels of apoptosis were similar between the two genders with liver tumors, and there was no significant increase of apoptosis in tumors compared to normal livers during early tumorigenesis (Fig. 2F–J).

The Wnt/β-catenin pathway is frequently deregulated in HCC and is an important contributing factor to tumorigenesis^19^. E-cadherin, a binding partner of β-catenin, also plays critical roles in liver tumorigenesis as a suppressor of tumor and invasion^20^. Our previous study has shown the gradual activation of WNT/β-catenin pathway through nuclear localization of β-catenin and loss of E-cadherin during *kras*-induced liver tumorigenesis^21^. In normal livers, β-catenin was localized in the cell membrane. In liver tumors, the nuclear staining of β-catenin was observed in both genders, with the males having a higher degree of β-catenin activation (Fig. 2M–O). Moreover, there was only partial loss of membrane E-cadherin in female liver tumors, whereas in male liver tumors there was almost complete loss of E-cadherin (Fig. 2R–T). Taken together, these observations suggested that the faster tumor progression in males might be contributed by the higher cell proliferation accompanied by a higher degree of β-catenin activation and complete loss of membrane E-cadherin.

### Male *kras* ^*V12*^ zebrafish developed more severe liver tumors than female *kras* ^*V12*^ zebrafish during long-term tumor induction

**Figure 3:**
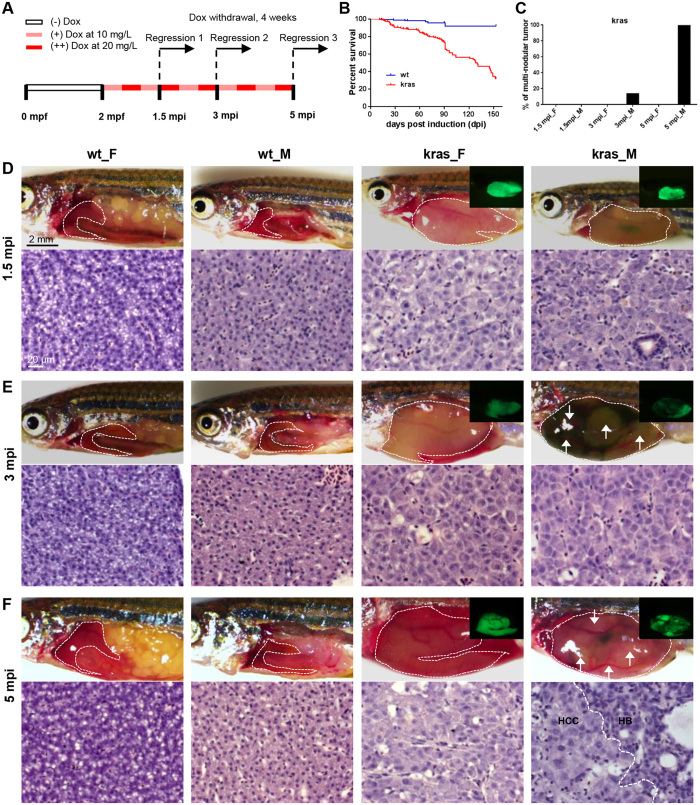
Male *kras*^*V12*^ zebrafish developed more severe liver tumors during long-term tumor induction. (**A**) Diagram of experimental design and schedules of sample collection for the long-term tumor induction. (**B**) Kaplan-Meier survival curve of wt and *kras*^*V12*^ fish. (**C**) Quantification of the percentage of multi-nodular tumors (n = 5–8 for each group). (**D**–**F**) Representative images and liver histology from female and male wild type and *kras*^*V12*^ fish at 1.5 mpi (**D**), 3 mpi (**E**) and 5 mpi (**F**). The livers are outlined in dash lines as well as marked by GFP expression in *kras*^*V12*^ fish. Multi-nodular tumor was observed only in male *kras*^*V12*^ fish at 3 mpi and 5 mpi. Individual nodules are indicated by GFP expression as shown in inlets for *kras*^*V12*^ fish and also pointed by arrows in the brightfield images. The multi-nodular tumors at 5 mpi were characterized by mixed types of HCC and HB separated by dash lines in the H&E staining. Scale bars, 2 mm for gross observation pictures and 20 μm for histology images.

We also performed a long-term tumor induction of the *kras*^*V12*^ zebrafish. Male and female zebrafish were treated with doxycycline and collected at 1.5 months post induction (mpi), 3 mpi and 5 mpi for examination of the tumor status, metastasis and ability of tumor regression after doxycycline removal. Alternative dosing of doxycycline at 10 and 20 mg/L was adopted to balance the tumor growth and fish survival (Fig. 3A). There was no difference of survival rate between female and male fish and the overall Kaplan-Meier survival curves of kras and wild type control are shown in Fig. 3B).

The gross appearance and histology of normal liver and tumor were presented in Fig. 3D–F. At 1.5 mpi, both male and female *kras*^*V12*^ fish developed homogeneous HCC. At 3 mpi, *kras*^*V12*^ male fish (14%, n = 7) began to develop multinodular HCC, while all the female liver tumors remained homogenous. By 5 mpi, all male *kras*^*V12*^ fish (100%, n = 8) developed more severe multinodular liver tumors, containing mixtures of hepatocellular adenoma (HCA), mixed HCC, and hepatoblastoma (HB). In comparison, the induced females still showed homogenous HCC. There were increasing cases of multinodular tumors in male *kras*^*V12*^ fish from 3 mpi (14%) to 5 mpi (100%) (Fig. 3C); however, no ectopic or metastatic tumor was observed during the five months of tumor growth in the *kras*^*V12*^ zebrafish.

**Figure 4:**
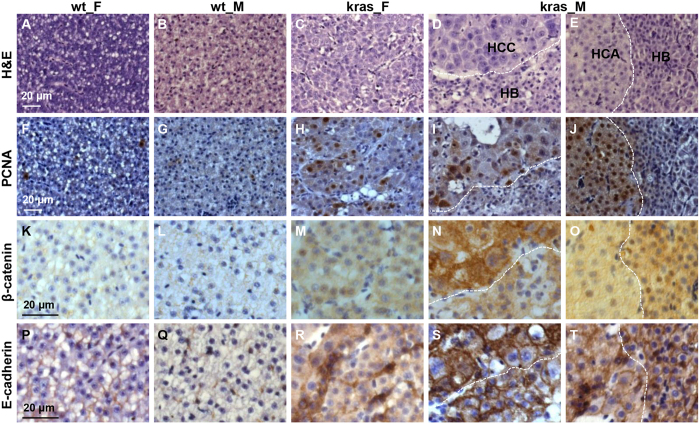
Characterization of the multi-nodular liver tumor from male *kras*^*V12*^ zebrafish after long-term tumor induction. Adjacent liver sections in female and male wild type and *kras*^*V12*^ fish at 5 mpi were processed for H&E and immunohistological staining. The multi-nodular male tumors have mixed types of tumors (HCC, HB and HCA) which were separated by dash lines. (**A**–**E**) H&E staining. (**F**–**J**) Immunocytochemical staining for PCNA. (**K**–**O**) Immunohistochemical staining for β-catenin. (**P**–**T**) Immunohistochemical staining for E-cadherin. Scale bar, 20 μm for all panels.

To investigate whether different nodules of male tumors were different in molecular characters, staining of PCNA, β-catenin and E-cadherin was carried out on wild type and *kras*^*V12*^ fish at 5 mpi (Fig. 4). The female tumors had generally uniform patterns of cell proliferation, nuclear internalization of β-catenin and loss of membrane E-cadherin (Fig. 4H,M,R). However, the multinodular male tumors showed highly uneven patterns of cell proliferation, β-catenin localization and membrane E-cadherin distribution among nodules or tumor types (Fig. 4I,N,S,J,O,T). These observations suggested the formation of different tumor colonies with differential morphological and molecular characteristics probably due to additional mutations. The cellular and membrane E-cadherin level was higher during long-term tumor progression than during early tumorigenesis (Figs 2R,S and 4R–T).

### The heterogeneous multi-nodular liver tumors of male *kras* ^*V12*^ zebrafish were also oncogene-addicted

Our previous study has shown that the induced HCC from *kras*^*V12*^ fish was dependent on Ras signaling for tumor maintenance, and withdrawal of inducer or suppression of *kras*^*V12*^ expression led to tumor regression^15^. The oncogene-addiction phenomenon was also observed in other inducible liver tumor lines driven by different oncogenes, including *xmrk* and *myc*^14^,^17^. However, additional mutation may occur during long-term tumor growth due to the genome instability and high mutation rate of tumor and thus it remains unknown whether the tumor was still dependent on *kras*^*V12*^ expression after prolonged period of tumor growth. Therefore, we next examined the ability of tumor regression after removal of doxycycline at different time points of the long-term tumor induction experiment as depicted in Fig. 3A.

**Figure 5:**
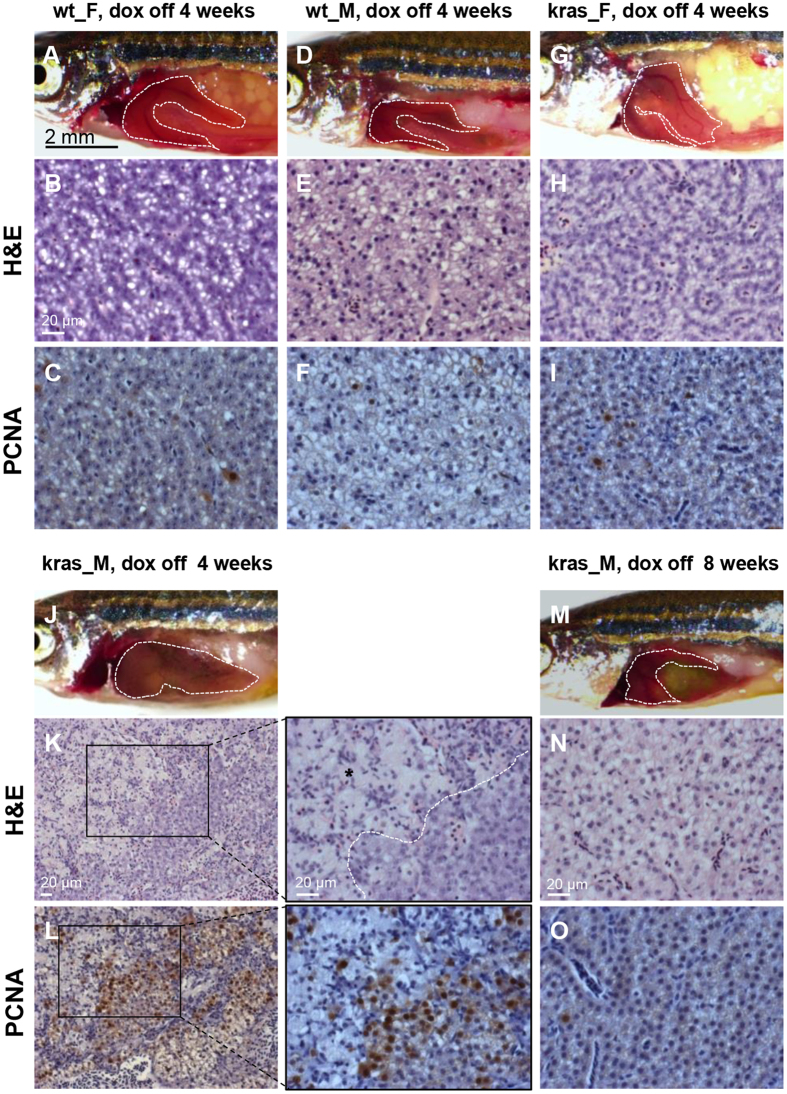
Withdrawal of doxycycline caused slower liver tumor regression in male *kras*^*V12*^ fish. Representative images, liver histology and PCNA immunostaining from female and male wild type and *kras*^*V12*^ fish after doxycycline withdrawal at 5 mpi are shown. (**A**–**C**) Gross observation (**A**), liver histology (**B**) and PCNA staining (**C**) of wt-F fish after 4 weeks of doxycycline withdrawal. (**D**–**F**) Gross observation (**D**), liver histology (**E**) and PCNA staining (**F**) of wt-M fish after 4 weeks of doxycycline withdrawal. (**G**–**I**) Gross observation (**G**), liver histology (**H**) and PCNA staining (**I**) of kras-F fish after 4 weeks of doxycycline withdrawal. Complete tumor regression in females after four weeks of doxycycline withdrawal were observed as indicated by the normal histology and PCNA staining. (**J**–**L**) Gross observation (**J**), liver histology (**K**) and PCNA staining (**L**) of kras-M fish after 4 weeks of doxycycline withdrawal. Incomplete tumor regression in males after four weeks of doxycycline withdrawal was indicated by the relatively larger liver size, extensive scar (marked by black asterisk) and high rate of PCNA-positive cells. (**M**–**O**) Gross observation (**M**), liver histology (**N**) and PCNA staining (**O**) of kras-M fish after 8 weeks of doxycycline withdrawal. Complete regression of male tumors after 8 weeks of doxycycline withdrawal. N = 5–8 for each group. Scale bars, 2 mm for gross observation pictures and 20 μm for histology images.

After tumor growth for 1.5 month, doxycycline withdrawal resulted in complete tumor regression within four weeks, as supported by both gross observation and histological analyses (Supplementary Fig. S1). After three months of induction, the liver tumors in both males and females were still reversible within four weeks of doxycycline withdrawal (Supplementary Fig. S1). By five months of induction, after doxycycline withdrawal, all the female livers showed complete tumor regression within 4 weeks, as indicated by the normal histology and cell proliferation (Fig. 5A–I). However, relatively larger liver size and extensive scar was present in all the male livers, accompanied by high level of cell proliferation (Fig. 5J–L). The histology and cell proliferation of male *kras*^*V12*^ liver returned to normal only after 8 weeks of doxycycline withdrawal (Fig. 5M–O). Hence, the time required for complete regression of male tumors was lengthened, but the multinodular, heterogeneous tumors of male were still oncogene-addicted.

### Sex hormone treatments affected the liver tumor progression in *kras* ^*V12*^ fish

**Figure 6:**
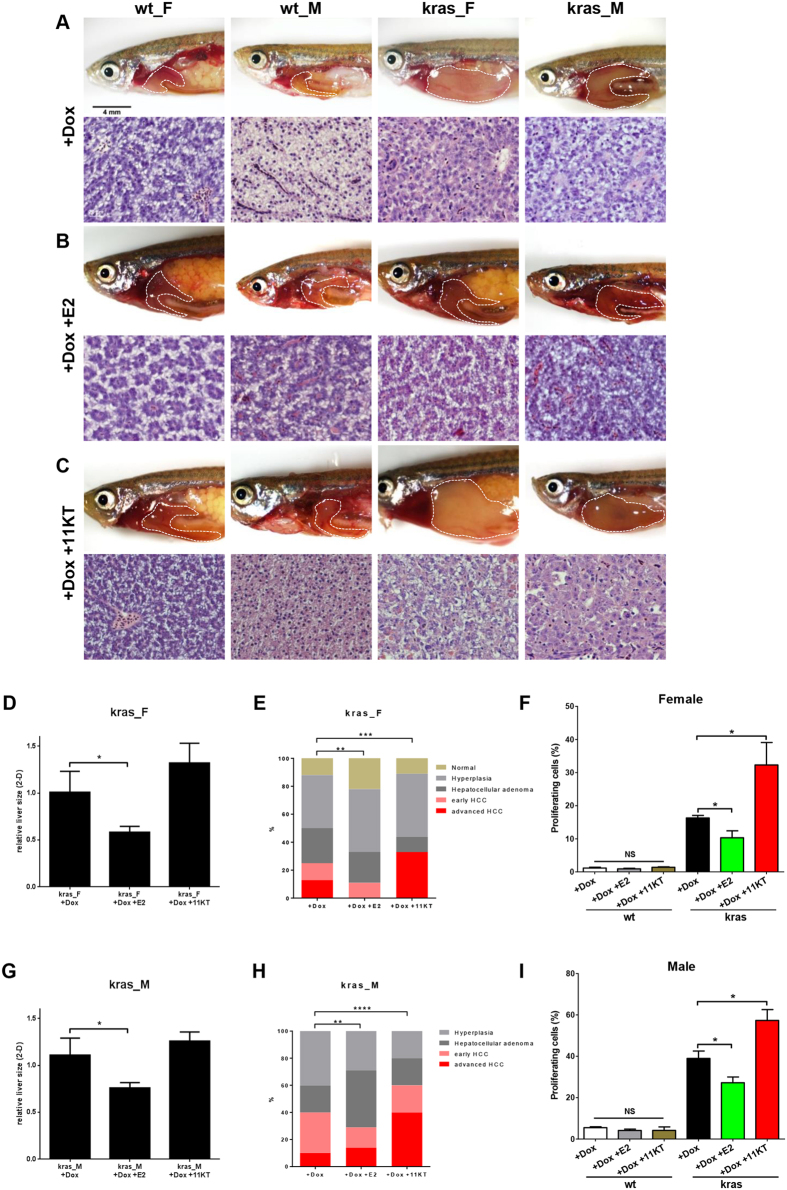
Sex hormone treatments affected cell proliferation during liver tumor progression in *kras*^*V12*^ fish. Wild type (wt) and *kras*^*V12*^ fish (4 months old) were treated with dox (doxycycline) alone, dox and E2, and dox and 11-KT, respectively, for a total of 7 days. (**A**–**C**) Representative gross observations and histology of wt and *kras*^*V12*^ fish after treatment with dox (**A**), dox and E2 (**B**), dox and 11-KT (**C**). (**D**–**F**) Relative 2D liver size (**D**), quantification of tumor phenotypes observed from the liver histology (**E**) and quantification of the proliferating cells (%) in different female treatment groups (**F**). (**G**–**I**) Relative 2D liver size (**G**), quantification of tumot phenotypes observed from the liver histology (**H**) and quantification of the proliferating cells (%) in different male treatment groups (**I**). N = 4–5 per group for wt, n = 7–10 per group for *kras*^*V12*^. *p < 0.05; **p < 0.01; ***p < 0.001; ****p < 0.0001; NS: not significant. Scale bars, 4 mm for gross observation pictures and 20 μm for histology images.

Since the female *kras*^*V12*^ fish developed HCC significantly slower and less aggressive than the male *kras*^*V12*^ fish under the identical experiment condition, our experiment system allowed us to further analysis the factors affecting the gender disparity in liver tumor. It has been reported that estrogen in females has protective roles during liver tumor progression while androgen in males accelerates the liver tumor development^22^,^23^. Thus, sex hormone treatments were performed to assess their roles in liver tumor progression. Adult *kras*^*V12*^ fish at 4 mpf were sexed. Male and female fish were treated with doxycycline in conjunction with 17-estrodiol (E2) or 11-ketotestosterone (11-KT), respectively. In preliminary experiments, we found that 7 day was the earliest time point to induce HCC phenotype in some doxycycline-treated *kras*^*V12*^ fish. Thus, after one week treatment, liver samples were collected for histological analyses. Representative gross observation and histology of normal liver and tumor are presented in Fig. 6A–C. After sex hormone treatment, in both male and female *kras*^*V12*^ adult fish, the relative tumor sizes showed significant decreases with E2 treatment and slight increases after 11-KT treatment (Fig. 6D,G). After doxycycline treatment for one week, 40% *kras*^*V12*^ male and 25% female fish showed characters of HCC including multiple nuclei, prominent nucleoli and abnormal mitosis; 20% males and 25% females of *kras*^*V12*^ adult fish showed the loss of the hepatic plate structure and high numbers of the hepatocytes, which are features of HCA; about 40% male and female *kras*^*V12*^ adult zebrafish showed liver hyperplasia. Furthermore, 12% female, but none of male, *kras*^*V12*^ fish showed normal histology after doxycycline induction for one week. In the presence of E2, the HCC ratios of the *kras*^*V12*^ male and female fish decreased to 29% and 11%, respectively; in the presence of 11-KT, the ratios of HCC increased to 60% and 33% in male and female *kras*^*V12*^ zebrafish, respectively (Fig. 6E,H). In addition, the structure of liver in male fish resembled the female fish after E2 treatment (Fig. 6B). Overall, histological analyses indicate that the E2 treatment deferred whereas 11-KT treatment accelerated the liver tumor progression in both male and female *kras*^*V12*^ fish.

### Sex hormone treatments affected cell proliferation during liver tumor progression in *kras* ^*V12*^ fish

To further investigate the effects of sex hormone treatments on liver tumor progression, PCNA staining was performed to examine the cell proliferation in liver after sex hormone treatments (Supplementary Fig. S2 and Fig. 6F,I). After E2 treatment, the PCNA-positive staining decreased dramatically in both male and female *kras*^*V12*^ fish, indicating the inhibition of cell proliferation in liver tumor by the estrogen E2 in both male and female *kras*^*V12*^ fish. After 11-KT treatment, in both male and female *kras*^*V12*^ fish the PCNA-positive cells increased significantly, suggesting that the androgen 11-KT enhanced tumor cell proliferation. The alternation of cell proliferation during liver tumor progression may be related to the effects of sex hormones on affecting liver tumor development: the estrogen inhibits cell proliferation during *kras*^*V12*^ liver tumor progression while the androgen promotes it. Meanwhile, in wild type male and female fish, neither E2 nor 11-KT had any effect on cell proliferation, suggesting that the dysfunction of cell proliferation in liver tumor might be the target of the sex hormone treatment.

## Discussion

Similar to that in mammals, the zebrafish liver is also a sex-dimorphic organ. This has been proven by comparison of the transcriptomic difference between female and male zebrafish^24^. In the present study, we demonstrated that the zebrafish liver tumor model could be a valuable tool for studying the gender disparity of HCC. There are several advantages of using the inducible *kras*^*V12*^ transgenic zebrafish model for studies of HCC gender disparity. First, HCC could be induced at any stage of life cycle as tumor initiation is determined by the timing of inducer treatment^15^. Second, there was dose-dependent induction of oncogene expression in the Tet-on inducible transgenic system and thus the speed of tumor development could be controlled^14^. Third, HCC could be induced as fast as one week using a relatively high dose of chemical inducer. Fourth, male and female *kras*^*V12*^ fish can be induced under identical experimental conditions for easy testing of variable factors contributing gender disparity of HCC. Furthermore, chemical screening could be feasibly performed using the zebrafish model to identity pathways that affect the gender disparity of HCC. We could also maintain the long-term progression of tumor without causing massive death of fish, by modulating the concentration of chemical inducer. Hence, the zebrafish liver tumor model is valuable for study of gender difference during early tumorigenesis as well as long-term tumor progression.

Oncogene addiction has been used to refer to the phenomenon that the malignant status is dependent on sustained activation of a specific oncogene^25^. The addiction to *kras*^*V12*^ has been reported in our previous study^15^. In the present study, we hypothesized that additional mutations could be accumulated with the progression of tumor and eventually made the tumor become independent of the expression of the primary *kras*^*V12*^ oncogene. For this purpose, we performed long-term tumor induction up to five months. It was not feasible to further extend the induction period, as there was massive death of fish after five months. Interestingly, all the males developed multi-nodular tumors containing mixed types of carcinoma, which may imply additional gene mutations and colony expansion. However, these multinodular tumors remained regressible, indicating a strong oncogene addiction to the primary *kras*^*V12*^ oncogene. It is possible that the five months of tumor induction was not sufficient to robustly produce another driver gene mutation, but our current experimental system does not allow us to extend a longer term of *kras*^*V12*^ expression because of a large mortality after 5 months of induction. Moreover, we cannot excluded the possibility that oncogene independency had emerged in a small group of cells, which was difficult to identify from the histological examination.

The gender disparity of HCC is an important topic in hepatocarcinogenesis. In human, women have significantly lower incidence and mortality of HCC than men do, in spite of the lifestyle differences between men and women, interestingly, women after menopause have higher possibility to develop HCC^22^, which indicates that the estrogen may have protective effects on HCC development and progression. To validate the protective effects of estrogen, we performed estrogen treatment on *kras*^*V12*^ adult fish and showed that the E2 treatment can defer the *kras*-driven liver tumor progression as indicated by histological analyses. Subsequent proliferation analyses confirmed that E2 treatment significantly inhibited cell proliferation during liver tumor progression, suggesting that the E2 treatment may interfere with the cell cycle regulation in liver tumor. E2 is the direct ligand of the estrogen receptor (ER), and the human ERα has been proved to protect HCC from tumor proliferation^26^. Furthermore, the male predominance of HCC also implicated the involvement of androgen and androgen receptor (AR) signaling. Human studies have shown that increased testosterone levels were significantly associated with the increased risk of HCC^27^. It has been reported that androgen/AR signaling could promote the early stage hepatocarcinogenesis, enhance cell growth by transcriptional regulation of TGFb1 and modulate cell cycle-related kinase transcription^28^. Consistent with these, in this study, we also showed that androgen treatment promoted liver tumor formation and increased cell proliferation *in vivo* using the zebrafish model.

Hormone therapy on cancer has been utilized clinically for many years, especially on some gender-related cancers such as prostate cancer and breast cancer^29^,^30^. It has been reported that hormone therapy also showed positive effects on liver cancer^31^; However, the efficacy of hormone therapy on the liver cancer remains controversial^32^,^33^. In the present study, we showed that E2 treatment inhibited liver tumor progression whereas 11-KT accelerated tumor growth. These results provide new evidence for hormone treatment of liver cancer and suggest that the zebrafish *kras*^*V12*^ HCC model could be used further for investigation of mechanisms of hormone treatment and screening other auxiliary factors in hormone treatments of HCC.

## Methods

### Zebrafish maintenance and chemical treatments

All zebrafish experiments were carried out in accordance with the recommendations in the Guide for the Care and Use of Laboratory Animals of the National Institutes of Health and the protocol was approved by the Institutional Animal Care and Use Committee (IACUC) of the National University of Singapore (Protocol Number: 096/12). *kras*^*V12*^ transgenic zebrafish *Tg(fabp10:rtTA2s-M2; TRE2:EGFP-kras*^*G12V*^) were previously generated^16^ and this transgenic line comprises a Tet-On system for inducible and liver-specific expression of oncogenic *kras*^*V12*^. Treatments of adult fish (4 months post-fertilization, mpf) were conducted in 6-liter tanks and water was changed every other day. For early tumorigenesis, *kras*^*V12*^ zebrafish and their wild type siblings were treated with 20 mg/l doxycycline (Sigma) for 10 days. For long-term tumor induction, pulse dosing of doxycycline was used (alternative use of 10 mg/l and 20 mg/l doxycycline bi-weekly) to maintain the tumor growth while reducing the death rate. For hormone treatments, 5 μg/l 17β-estradiol (E2) (Sigma) or 11-ketotestosterone (11-KT) was used in conjunction with 20 mg/l doxycycline for 7 days.

### Paraffin sectioning and histological analysis

Fish abdominal regions were cut and fixed in formalin solution (Sigma-Aldrich), dehydrated and embedded into paraffin. Sections at 5 μm thickness were processed using a microtome. These sections were deparaffinized, rehydrated and stained with Mayer’s hematoxylin (Vector Laboratories) and eosin (Sigma-Aldrich). The stained slides were mounted in Micromount (Leica) and imaged using an inverted light microscope (Zeiss, Axiovert 200 M). Classification of tumor types were based on established criteria as previously reported^34^. For hyperplasia, the major criteria are maintenance of hepatic plates, large nuclei with occasionally prominent nucleoli. For HA, the major criteria are loss of hepatic plates, large but uniform nuclei, rare mitosis. For HCC, the criteria are loss of hepatocyte cell plates, large and variable nuclear size, prominent nucleoli and the presence of multiple nucleoli. For HB, the cells are embryonal, small and more basophilic, have scant cytoplasm and large nuclei with prominent nucleoli.

### Immunohistochemistry staining and TUNEL assay

For immunohistochemistry, the paraffin sections were deparaffinized and rehydrated. Antigen retrieval was performed using sodium citrate buffer by heating in a boiling water bath for 20 min. Sections were then treated with 3% H_2_O_2_ for 10 min to inhibit the endogenous peroxidase activity. After blocking, primary antibodies were incubated at 4 °C for overnight. HRP (horseradish peroxidase)-conjugated secondary antibodies were incubated at room temperature for one hour, followed by color development using the DAKO Real Envision Detection System. The primary antibodies used included rabbit anti-PCNA 1:500 (Santa Cruz), rabbit anti-β-catenin 1:250 (Abcam) and mouse anti-E-cadherin 1:250 (BD Biosciences). TUNEL assay was performed using the *In Situ* Cell Death Detection kit (Roche) according to the manufacturer’s instruction.

### Statistical analyses

Statistical significance between two groups was evaluated by two-tailed unpaired Student t test and Chi-square test (for tumor histological classification only) (GraphPad). Statistical data were presented as mean value ± standard error of mean (SEM). P < 0.05 was chosen to be statistically significant.

## Additional Information

**How to cite this article:** Li, Y. *et al*. Males develop faster and more severe hepatocellular carcinoma than females in **kras^V12^** transgenic zebrafish. *Sci. Rep.* **7**, 41280; doi: 10.1038/srep41280 (2017).

**Publisher's note:** Springer Nature remains neutral with regard to jurisdictional claims in published maps and institutional affiliations.

## Supplementary Material

Supplementary Figures
