# Supplementary material for: Males develop faster and more severe hepatocellular carcinoma than females in krasV12 transgenic zebrafish
Source: Sci Rep. 2017 Jan 24;7:41280. doi: 10.1038/srep41280 (PMC5259773; doi:10.1038/srep41280)
Supplement: Supplementary Figures [file srep41280-s1.pdf]

Supplementary Information

Males develop faster and more severe hepatocellular carcinoma than females in *kras*^{V12} transgenic zebrafish

Yan Li[1,3], Hankun Li[1,3], Jan M. Spitsbergen[2], Zhiyuan Gong[1,] *

[1]Department of Biological Sciences, National University of Singapore, Singapore 117543.

[2]Department of Microbiology, Oregon State University, Corvallis, Oregon, USA, 97331.

[3]These authors contributed equally.

* Authors for correspondence (dbsgzy@nus.edu.sg)

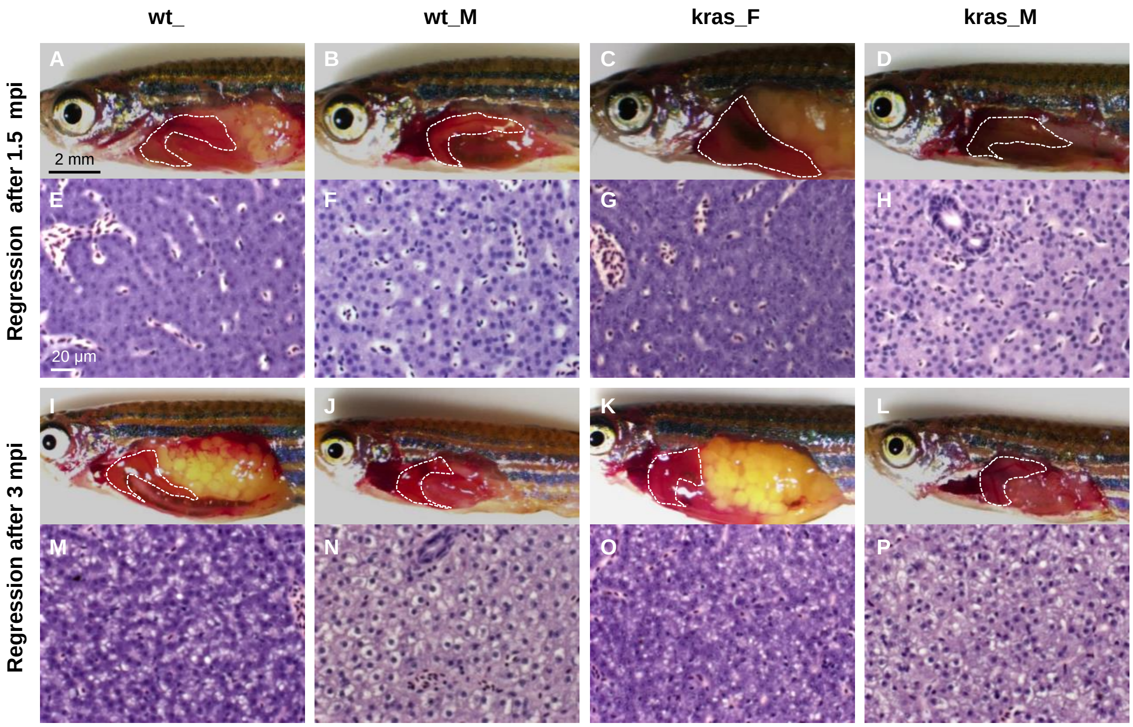

Supplementary Figure S1. Tumor regression at 1.5 mpi and 3 mpi after four weeks of doxycycline withdrawal. Representative images and liver histology from female and male wild type and *kras*^{V12} fish are shown. (A-H) Liver morphology (A-D) and histology (E-H) after four weeks of doxycycline withdrawal at 1.5 mpi. (I-P). Liver morphology (I-L) and histology (M-P) after four weeks of doxycycline withdrawal at 3 mpi. Complete tumor regression was observed in in all the female and male *kras*^{V12} fish, indicated by normal liver size and histology. Scale bars: 2 mm in (A-D) and (I-L); 20 µm in (E-H) and (M-P).

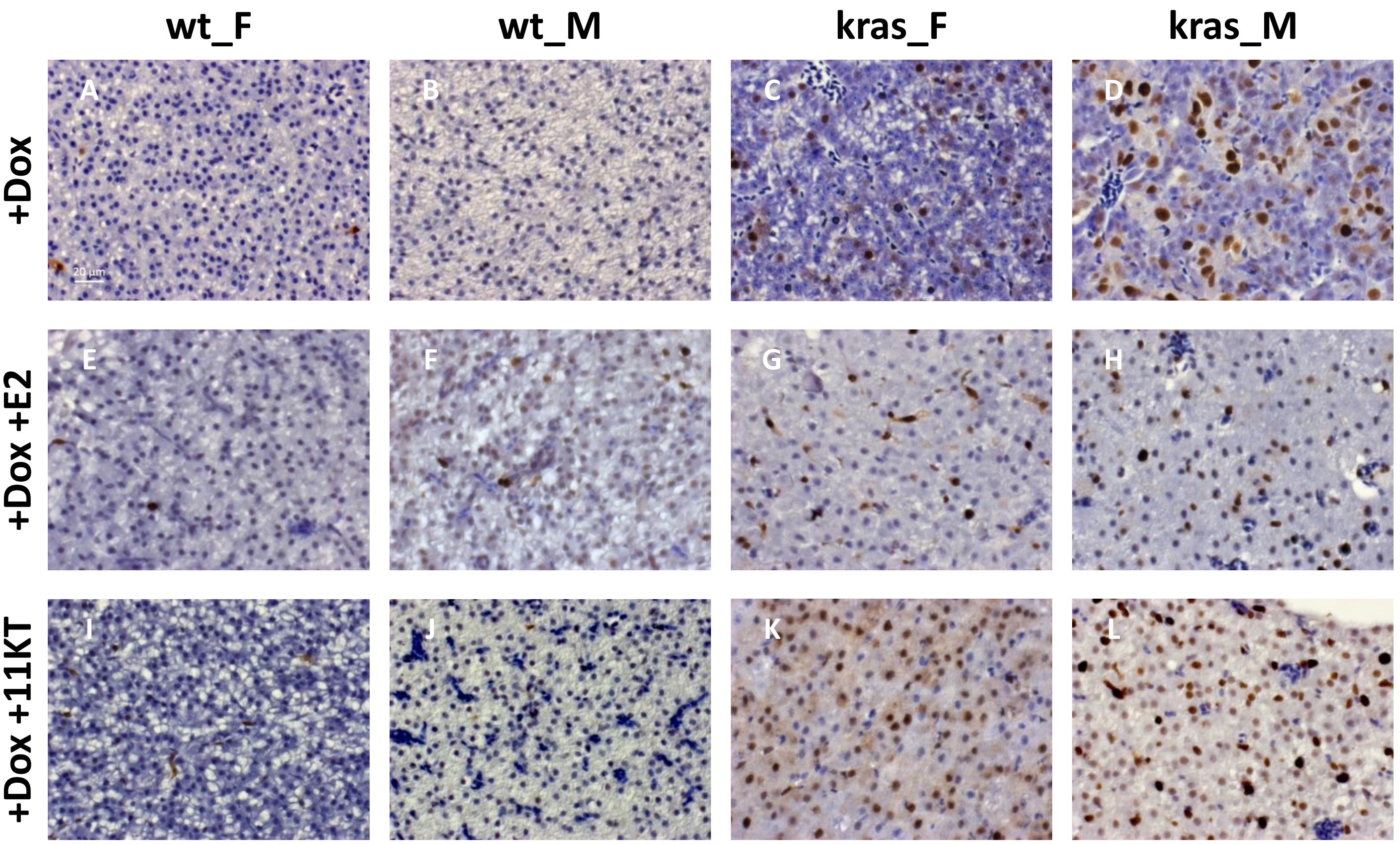

Supplementary Figure S2. Effects of sex hormones on cell proliferation during liver tumor progression in adult *kras*^{V12} zebrafish. Wild type (wt) and *kras*^{V12} fish (4 months old) were treated with dox (doxycycline) alone, dox and E2, and dox and 11-KT, respectively, for a total of 7 days. Liver sections were stained for PCNA. (A-L) Representative images of PCNA staining of female and male wt and *kras*^{V12} liver after treatments of dox (A-D), dox and E2 treatment (E-H), or dox and 11-KT (I-L). Scale bar, 20 µm for all panels.
